# From a Neglected Pathogen to a Public Health Emergency: Connecting the Dots in Monkeypox Emergence

**DOI:** 10.7759/cureus.28667

**Published:** 2022-09-01

**Authors:** Monica Fahmy, Lina Shabata, Yahia Aktham

**Affiliations:** 1 Wolfson Institute of Population Health, Queen Mary University of London, London, GBR; 2 Medical Affairs, Sanofi, Kuwait, KWT; 3 Medical Affairs, Sanofi, Jeddah, SAU

**Keywords:** mpx, outbreak, emerging infectious diseases, monkeypox, mpxv

## Abstract

The recent unprecedented emergence of the monkeypox (MPX) outbreak rang an alarm bell creating a burden on global health. The virus historically spread in endemic regions of sub-Saharan Africa by animal-human and human-human modes of transmission with an unknown modality. Currently, cases are occurring worldwide while risk factors, transmission, and outcomes of infection are still poorly defined. In addition, the likely genotypic mutation of MPX is an area of concern and requires further assessment. While licensed smallpox vaccinations are currently considered for early prevention after recent exposure, and antivirals are recommended for treating severe cases, there are no treatments available to treat MPX. This review provides general disease awareness and highlights recommendations for the containment of the virus.

## Introduction and background

The first discovery of monkeypox (MPX) was in 1958 when two outbreaks occurred in colonies of monkeys and looked like pox disease in a Danish laboratory [1,2]. However, monkeys are not the source of infection, as rodents such as squirrels and Gambian pouched rats have had the largest reservoirs of the virus [3,4]. The first human case was reported in the Democratic Republic of Congo in 1970 when the virus was detected in persons living in remote African locations followed by the 1980s, which had a growing number of monkeypox cases in humans, with a total of 63 deaths from the 1970s to 1990, and a subsequent major outbreak during 1996-1997, leading to 181 clinical cases, including nine deaths in multiple African countries at the same time when efforts were intensified to eradicate smallpox [5,6]. Since then, 10 other African countries have reported infected cases [2,7]. In 2003, the first outbreak in the USA was linked to contact with infected pet dogs, which had been housed with Gambian pouched rats imported from Ghana [7]. The case was a three-year-old girl hospitalized with cellulitis and fever after a bite by a pet dog [7]. Surprisingly, the dog also became ill on the same day of the bite and died seven days later, and the girl’s mother died in the same month with evidence showing electron-microscopic poxvirus in a skin lesion [7]. This was followed by an outbreak of 47 cases of monkeypox in the United State of America (USA) [2,7,8].

## Review

Epidemiology

Between May 6 and August 3, 2022, the world reported 26,048 confirmed cases of MPX, including 10 deaths, The 10 fatal cases were reported in Brazil (1), India (1), Nigeria (3), Peru (1), Spain (2), and the Central African Republic (CAR) (2) [9]. Reported cases were in countries where the disease is not endemic, with the majority of cases not traveling to endemic areas of western or central Africa but currently spreading in widely distributed geographical regions [10]. Researchers indicated that smallpox vaccination with vaccinia virus was around 85% protective against monkeypox because 10 CAR MPX genomes have the typical organization observed in other orthopoxviruses, which include the smallpox virus [11]. The reported data from the previous outbreaks also showed that the attack rate of MPX was approximately 80-96% lower among individuals with a history of prior smallpox vaccination compared to unvaccinated individuals and the highest percentage of vaccinated cases (21%) was found in the US outbreak [6]. Therefore, the surge in the cases and duration of monkeypox epidemics could be a result of the decline in smallpox vaccination due to the eradication of the disease in 1980 [6,12]. Several epidemiological data stated that infection rates are disproportionally higher in males than females, which calls for vigilance in surveillance and public health efforts [13-15]. MPX has been labeled a neglected tropical disease since the early ’70s; as a result, the emergence in many non-endemic regions is likely to be the outcome of ignorance [16].

Virology

Monkeypox virus (MPXV) is a double-stranded DNA zoonotic virus that is a member of the Poxviridae family, genus Orthopoxvirus, known to include the causative agent of smallpox, variola virus, and vaccinia virus [17]. MPX is a void or brick-shaped particle covered by lipoprotein outer membrane sizes between 200 and 250 nm due to the large size of MPXV, which makes it harder to breach host defenses by passing through gap junctions reflecting the difficulty for the virus to replicate rapidly [14,18]. Within the cytoplasmic structure of the virus is the DNA replication, gene expression, and mature virions assembly. However, these particles remain intracellular until they are transported to the Golgi compartment, after which they are wrapped with two more lipid bilayers and generate extracellular viruses (EVs) [17]. These are responsible for cell-to-cell spread at long distances and are the reason for animal pathogenicity; thus, the host proteins are essential to complete the viral lifecycle of MPX, like other poxviruses [17]. For a better understanding of host-pathogen interactions, scientists identified host factors required for infection. These factors are components of the Golgi-associated retrograde protein (GARP) complex, vacuolar protein sorting-associated protein 52 (VPS52), and vacuolar protein sorting-associated protein 54 (VPS54) proteins [17]. GARP is one of the several multi-subunit complexes that are part of the vesical recognition for the MPX virus membrane fusion and contains four proteins present in an equal ratio [17].

Mode of transmission

There are two main modes of transmission of the disease: animal to human and human to human. It was suspected in several studies that animal consumption, hunting, and handling were possible ways of transmitting the disease from animals to humans, but these were not definitive, making MPX a complex transmission puzzle [5,15]. Other researchers have noticed nosocomial outbreaks due to aerosol transmissions. Nevertheless, contact with living or dead animals is probably the driver of human MPX infections, manifested in bodily fluids such as saliva, respiratory tract secretions, exudates from cutaneous or mucosal lesions, or exposure to feces [3,14]. On the other hand, human-to-human transmission involves but is not limited to respiratory droplets due to prolonged face-to-face contact, exposure to lesions of infected individuals, or exposure to contaminated surfaces [14]. While the World Health Organization (WHO) has not yet confirmed whether MPX is a sexually transmitted disease, reports showed that locations of symptoms were mainly in the anogenital and perineal areas and involved inguinal lymphadenopathy, suggesting that this could be a key role in transmission [3,14,15]. MPX rarely spreads asymptomatically; however, the recent outbreak in around 68 countries raises concerns about the potential genotypic mutations that could be changing the virus phenotype, which could be a sign of either increased virus transmissibility or a slow progression that is hard to track [19].

Clinical manifestations

Following the viral entry to the human body through any of the routes, it starts to replicate at the site of inoculation, from which the viral load spreads to the local lymph nodes in the primary viremia and the distant lymph nodes and organs through circulation in the secondary viremia [14]. This pathway resembles smallpox, and the incubation period is estimated between five and 21 days without visible clinical symptoms, suggesting that the incubation period is not contagious [3,14]. The total duration of signs and symptoms takes two to five weeks starting with the initial febrile prodrome stage, which comprises non-specific signs and symptoms of fever, headache, cough, general fatigue, back pain, myalgia, and lymphadenopathy; these signs and symptoms happen before skin rash of the face and extremities, which occurs one to five days after the onset of fever [3,14,15]. Lymphadenopathy is not a characteristic of smallpox, and it is developed in the maxillary, cervical, and/or inguinal areas, which appear enlarged, firm, and painful [20]. Rash often appears first on the face, followed by a quick centrifugal distribution on the body, described as more lesions on extremities than on the abdomen and trunk [14,20]. Over the course of two to four weeks, the lesions synchronously change from macular, popular, vesicular to pustular phases. They are often seen as erythematous and/or skin hyperpigmented areas around discrete lesions along with pharyngeal, conjunctival, and genital mucosae inflammation [3,14,21,22].

Crust starts to form five to seven days after the pustular phase of the lesions, and they desquamate between one to two weeks. The course of the disease concludes in a total duration of three to four weeks. Patients become non-infectious after the crusts are all off, making MPX a self-limiting disease [7,14,21,23]. Higher rates of secondary bacterial infections have been reported in unvaccinated monkeypox patients, and other complications of respiratory distress, bronchopneumonia, encephalitis, vision loss, gastrointestinal involvement, vomiting, diarrhea with dehydration, and septicemia when lesions count are >4500 have also been observed [3,20]. Severe cases usually occur among children and immunocompromised individuals. Generally, younger patients are more susceptible to the disease due to the cessation of smallpox vaccination after the historical eradication of the outbreak [7]. Case fatality rates are distinct according to the clades of MPX, with the Central African clade having a poorer prognosis than the West African clade of 11% versus the 1% case fatality rate in unvaccinated children, respectively [21]. Recent data based on PCR assays indicate that the ongoing MPX outbreak belongs to the West African clade [24]. Although monkeypox clinical presentation is mild and of a lower-case fatality rate than smallpox, delayed treatment can lead to a fatality rate approaching 10%, and pulmonary failure is reported as one of the most common fatal symptoms [18,19]. On July 23, 2022, the ongoing MPX outbreak was declared a Public Health Emergency of International Concern, a term used by WHO to define an extraordinary event that carries cross-border implications for public health and requires immediate international action [25].

Evaluation and diagnosis

Detailed medical history is pivotal in diagnosis by including information on recent travel to endemic areas, previous smallpox immunization, and clinical picture. However, the gold standard for detecting MPX DNA is the real-time polymerase chain reaction (PCR) test owing to its sensitivity and accuracy; thus, the PCR test should be done before other diagnostic assays [26]. Specimens for diagnostic testing include the roof of vesicles' fluid and pustules of skin lesions and dry crusts [27]. At least two crusts or materials from vesicles should be collected in separate sterile containers [27]. The diagnostic techniques include four methods as detailed below:

Genetic Method

This includes PCR and quantitative PCR (qPCR) and is based on nucleic acid amplification tests. The viral DNA in the lesion is stable for a considerable period if kept in a dark, cool environment. Yet, the disadvantage of this method is the high cost of equipment and reagents, which limits its utility in resource-deprived areas [28]. In addition, the Food and Drug Administration (FDA)-cleared PCR test for monkeypox collects specimens from infected lesions, which hinders the early diagnosis during the incubation period [7].

Phenotypic Method

This is based on clinical signs and symptoms. It is essential to look for lymphadenopathy because it’s a differential sign from other diseases [28]. Despite the high sensitivity of this method, which is between 93-98%, it has low specificity of 9-26%, which can lead to more false-positive results [28].

Immunological Method

This is antibody-based diagnosing that has cross-reactivity with other Orthopoxviruses, which makes this test valuable only when there is a previous indication to explain the causality of the disease [28]. immunoglobulin M (IgM) is more effective than IgG in diagnosing newly infected patients [5,28].

Electron Microscopy

This is not a specific test but can distinguish Orthopoxviruses from Herpesviridae. However, it cannot morphologically distinguish MPX [29].

Clinical management

Despite the lack of MPX treatment to date, the Center for Diseases Control and Prevention (CDC) recommends vaccination within four days of disease exposure to prevent the onset and within 14 days to reduce the severity [21]. Ankara vaccine, a replication-defective modified vaccinia virus, is given as a two-shot series, a month apart, and has proven to have a higher safety profile than the first and second generations of smallpox vaccines. Due to being attenuated, it is safe for atopic and immuno-compromised patients who are contraindicated from live-viruses administration [21]. Supportive medicines, such as antipyretics, analgesics, and antibiotics, may be included [26]. Furthermore, isolation, surgical masks, and coverage of lesions are necessary practices [21]. Antivirals that are approved for smallpox are being used without supporting evidence for MPX, such as Brincidofovir (200 mg/week orally), FDA approved in 2021, and Tecovirimant (600 mg twice daily for 2 weeks orally), FDA approved in 2018; however, the former has higher toxic side effects, including nephrotoxicity, cytomegalovirus, transaminitis, and nausea. Therefore, the latter is preferred [27,29]. Another promising antiviral therapy is Cidofovir, a viral DNA-polymerase inhibitor licensed for use in cytomegalovirus retinitis in acquired immune deficiency syndrome (AIDS) patients. CMX-001 is a modified cidofovir under development that lacks nephrotoxicity and can be administered orally. ST-246 is an oral inhibitor of intracellular virus release and is currently under an investigational protocol in the USA [29].

## Conclusions

The current MPX outbreak is clearly an international public health problem, A comprehensive understanding of the epidemiological characteristics of MPX and disease pathology is the first step in formulating the prevention measures. The decrease in population immunity associated with the discontinuation of smallpox vaccination has established the landscape for the reappearance of monkeypox. This is demonstrated by the increases in the number of cases and the median age of individuals acquiring monkeypox as well as the re-emergence of outbreaks in some countries after an absence of 30-40 years. Further, the appearance of cases outside of Africa highlights the risk for geographical spread and the global relevance of the disease. Countries around the world should increase attention to disease surveillance systems and scale up country readiness and response operations for rapidly identifying new cases of monkeypox, as well as put more effort into vaccine developments and expedite the ongoing research for potential antiviral treatments. It is also imperative to study the different behaviors of individuals in other endemic and non-endemic regions closely because, in the era of globalization, social determinants of health have historically played roles in the spread of diseases, particularly measures related to poverty, socio-economic status, access to healthcare, sanitation, and potentially sexual behaviors. These determinants form a vicious cycle and require a collective partnership of global and regional leaders to contextualize a roadmap for responding to the emergence of neglected tropical diseases. The efforts should not only focus on creating robust surveillance systems or promoting health by re-introducing smallpox as a mandatory vaccination but should also implement poverty alleviation strategies and invest in the poorly resourced regions of the world.

## References

[1] Magnus P von, Andersen EK, Petersen KB, Birch-Andersen A (1959). A pox-like disease in cynomolgus monkeys. Acta Pathol Microbiol Scand A.

[2] (2022). Monkeypox. National Foundation for Infectious Diseases. https://www.nfid.org/infectious-diseases/monkeypox/.

[3] Petersen E, Kantele A, Koopmans M, Asogun D, Yinka-Ogunleye A, Ihekweazu C, Zumla A (2019). Human monkeypox: epidemiologic and clinical characteristics, diagnosis, and prevention. Infect Dis Clin North Am.

[4] Radonić A, Metzger S, Dabrowski PW (2014). Fatal monkeypox in wild-living sooty mangabey, Côte d'Ivoire, 2012. Emerg Infect Dis.

[5] Beer EM, Rao VB (2019). A systematic review of the epidemiology of human monkeypox outbreaks and implications for outbreak strategy. PLoS Negl Trop Dis.

[6] Bunge EM, Hoet B, Chen L, Lienert F, Weidenthaler H, Baer LR, Steffen R (2022). The changing epidemiology of human monkeypox—a potential threat? A systematic review. PLoS Negl Trop Dis.

[7] (2022). Monkeypox. https://www.who.int/news-room/fact-sheets/detail/monkeypox.

[8] Mahase E (2022). Monkeypox: what do we know about the outbreaks in Europe and North America?. BMJ.

[9] (2022). Monkeypox. Our world in data. https://ourworldindata.org/monkeypox.

[10] Jang YR, Lee M, Shin H (2022). The first case of monkeypox in the Republic of Korea. J Korean Med Sci.

[11] Berthet N, Descorps-Declère S, Besombes C (2021). Genomic history of human monkey pox infections in the Central African Republic between 2001 and 2018. Sci Rep.

[12] Fine PE, Jezek Z, Grab B, Dixon H (1988). The transmission potential of monkeypox virus in human populations. Int J Epidemiol.

[13] Wallau GL, Maciel-de-Freitas R, Schmidt-Chanasit J (2022). An unfolding monkeypox outbreak in Europe and beyond. Mil Med Res.

[14] Kaler J, Hussain A, Flores G, Kheiri S, Desrosiers D (2022). Monkeypox: a comprehensive review of transmission, pathogenesis, and manifestation. Cureus.

[15] Peiró-Mestres A, Fuertes I, Camprubí-Ferrer D (2022). Frequent detection of monkeypox virus DNA in saliva, semen, and other clinical samples from 12 patients, Barcelona, Spain, May to June 2022. Euro Surveill.

[16] Tambo E, Al-Nazawi AM (2022). Combating the global spread of poverty-related Monkeypox outbreaks and beyond. Infect Dis Poverty.

[17] Realegeno S, Puschnik AS, Kumar A (2017). Monkeypox virus host factor screen using haploid cells identifies essential role of GARP complex in extracellular virus formation. J Virol.

[18] Kumar S, Subramaniam G, Karuppanan K (2022). Human monkeypox outbreak in 2022. J Med Virol.

[19] Farahat RA, Abdelaal A, Shah J (2022). Monkeypox outbreaks during COVID-19 pandemic: are we looking at an independent phenomenon or an overlapping pandemic?. Ann Clin Microbiol Antimicrob.

[20] Macneil A, Reynolds MG, Braden Z (2009). Transmission of atypical varicella-zoster virus infections involving palm and sole manifestations in an area with monkeypox endemicity. Clin Infect Dis.

[21] Moore MJ, Rathish B, Zahra F (2022). Monkeypox. StatPearls Publishing.

[22] Ligon BL (2004). Monkeypox: a review of the history and emergence in the Western hemisphere. Semin Pediatr Infect Dis.

[23] Weaver JR, Isaacs SN (2008). Monkeypox virus and insights into its immunomodulatory proteins. Immunol Rev.

[24] (2022). Multi-country monkeypox outbreak: situation update. https://www.who.int/emergencies/disease-outbreak-news/item/2022-DON396.

[25] (2022). WHO Director-General declares the ongoing monkeypox outbreak a Public Health Emergency of International Concern. Accessed July 26, 2022. https://www.who.int/europe/news/item/23-07-2022-who-director-general-declares-the-ongoing-monkeypox-outbreak-a-public-health-event-of-international-concern.

[26] Cheema AY, Ogedegbe OJ, Munir M, Alugba G, Ojo TK (2022). Monkeypox: a review of clinical features, diagnosis, and treatment. Cureus.

[27] Quarleri J, Delpino MV, Galvan V (2022). Monkeypox: considerations for the understanding and containment of the current outbreak in non-endemic countries. Geroscience.

[28] Hraib M, Jouni S, Albitar MM, Alaidi S, Alshehabi Z (2022). The outbreak of monkeypox 2022: an overview. Ann Med Surg (Lond).

[29] McCollum AM, Damon IK (2014). Human monkeypox. Clin Infect Dis.

